# Metabolic and endocrine profiles and hepatic gene expression of Holstein cows fed total mixed ration or pasture with different grazing strategies during early lactation

**DOI:** 10.1186/s13028-015-0163-6

**Published:** 2015-10-16

**Authors:** Ana Laura Astessiano, Ana Meikle, Maite Fajardo, Jorge Gil, Diego Antonio Mattiauda, Pablo Chilibroste, Mariana Carriquiry

**Affiliations:** Department of Animal Production and Pastures, School of Agronomy, Universidad de la República (UdelaR), Av. E. Garzón 780, C.P. 12900 Montevideo, Uruguay; Laboratory of Nuclear Techniques, School of Veterinary Medicine, Universidad de la República (UdelaR), C/Lasplaces 1550, C.P. 11600 Montevideo, Uruguay; Department of Health in Livestock Systems, School of Veterinary Medicine, Universidad de la República (UdelaR), EEMAC, Paysandú, C.P. 6000 Uruguay

## Abstract

**Background:**

In dairy mixed production systems, maximizing pasture intake and total mixed ration (TMR) supplementation are management tools used to increase dry matter and energy intake in early lactation. The objective was to evaluate metabolic and endocrine profiles and hepatic gene expression of Holstein cows fed either TMR ad libitum (without grazing) or diets combining TMR (50 % ad libitum DM intake) and pasture with different grazing strategies (6 h in one grazing session or 9 h in two grazing sessions) in early lactation. Pluriparous cows were grouped by calving date, blocked within group by body weight and body condition score (BCS) and randomly assigned to one of three feeding strategies from calving (day 0) to 60 days postpartum: control cows fed TMR ad libitum (G0; confined cows fed 100 % TMR without access to pasture), pasture grazing with 6 h of access in one session supplemented with 50 % TMR (G1), and 9 h of access in two sessions supplemented with 50 % TMR (G2).

**Results:**

Net energy (NE), but not metabolizable protein (MP), demands for maintenance and/or milk increased in G2 when compared with G1 and G0 cows, respectively. However, NE and MP balances were lower in G1 and G2 than G0 cows. Cow BCS at +55 days was greater in G0 than G2 cows and probability of cows cycling during the first month was greater in G0 and G1 than G2 cows. During the postpartum period, non-esterified fatty acids were greater in G1 than G2 and G0 and β-hydroxybutyrate was greater in G1 and G2 than G0 cows. Plasma insulin was greater and insulin-like growth factor (IGF)-I tended to be greater in G0 than G2 cows, leptin was greater in G2 and G0 and adiponectin were greater in G2 cows. Hepatic expression of growth hormonereceptor-*1A* and *IGF1* mRNA decreased while *IGF binding proteins 1,2,4,5* and *6* (*IGFBP*) mRNA as well as mRNA expression of insulin, leptin (*LEPRb)* and adiponectin-2 receptors increased from pre to postpartum in all cows. However, only hepatic *IGFBP6* and *LEPRb* mRNA were greater in G2 than G0 and G1 cows, respectively.

**Conclusion:**

Metabolic-endocrine profiles of cows with different feeding strategies in early lactation reflected not only changes in milk energy output and energy balance but also in walking and grazing activity. Concentrations of insulin and IGF-I were increased in G0 cows whereas plasma adiponectin and both, insulin and leptin sensitivity were improved G2 cows. Increased NE demands in G2 cows when compared to G1 and G0 cows, implied a metabolic stress that impacted negatively on reproductive function.

**Electronic supplementary material:**

The online version of this article (doi:10.1186/s13028-015-0163-6) contains supplementary material, which is available to authorized users.

## Background

Pasture-based dairy production systems have gained interest during the last decade due to their economic, environmental and animal-welfare advantages [1]. However, these production systems do not allow cows to approach their genetic potential for milk production and dry matter intake (DMI), especially in early lactation [2]. Maximizing DMI is crucial for a successful establishment of high lactation as the onset of lactation is accompanied by a period of negative energy balance during which, the high-producing dairy cows mobilize body energy reserves and lose body condition score (BCS) to support the copious amount of milk produced [3, 4].

The transition between late pregnancy and early lactation involves coordination among organs and tissues, including the mammary gland and metabolically active tissues like liver and adipose tissue. This is achievable, among other mechanisms, because the liver becomes refractory to growth hormone (GH) uncoupling the somatotrophic axis. The uncoupling of the GH-IGF axis is associated to a catabolic endocrine state of increased GH and decreased IGF-I concentrations that, together with low concentrations of insulin, support tissue mobilization, increased liver gluconeogenesis, and high peak milk production [3, 5]. In addition, adipose tissue plays its role not only in the storage and mobilization of lipids but also as an active endocrine tissue sensing metabolic signals and secreting hormones (i.e. leptin and adiponectin) that affect whole-body energy homeostasis through modulation of glucose and fatty acids metabolism in peripheral tissues, and central regulation of feed intake and energy expenditure [6–8]. Improving nutrition of the postpartum dairy cow will not only impact on milk production but also will minimize the negative effects of the catabolic situation (i.e. IGF-I and insulin) on reproductive performance [9].

In grazing dairy cows, it has been reported that energy intake is the primary determinant of milk production. Thus, most grazing systems incorporate supplementary feeds in the form of forage and concentrates and more recently as mixed rations [10]. Supplementing grazing dairy cows with mixed rations has the potential to capitalize on the benefits of formulated total mixed rations (TMR) while maintaining a relatively low-cost feeding system based on grazed pasture. Previous research has demonstrated that high levels of supplement, as TMR, increased DMI and milk production of grazing dairy cows [10].

Herbage intake in grazing dairy cows is not only limited by physiological and behavioral constraints but also by sward characteristics and grazing management constraints [10, 11]. Previous reports indicated that daily herbage allowance had a major role in milk production and/or days to first ovulation in supplemented primiparous cows in early lactation, although the very low proportion of the allowed grazing time is spent grazing [9, 12]. Thus, daily access time to pasture and number of grazing sessions could be also used as grazing management tool to improve pasture DMI and milk production, as well as to increase grazing efficiency in dairy cows [11].

The hypothesis of this work was that TMR feeding will result in greater milk production and better reproductive performance than grazing dairy cows, and that the increase of the daily access time at pasture together with the number of grazing sessions during the first 60 days of lactation will maximize pasture DMI and improve energy balance of dairy cows in early lactation. The aim of the present study was to evaluate metabolic and endocrine profiles and hepatic gene expression of Holstein cows fed either TMR ad libitum (without grazing) or diets combining TMR (50 % ad libitum DM intake) and pasture with different grazing strategies (6 h in one grazing session or 9 h in two grazing sessions) in early lactation.

## Methods

The experiment was carried out at the Experimental Station “Dr. Mario A. Cassinoni” of the School of Agronomy (EEMAC, Paysandú, Uruguay) from March to June 2011. Animal procedures were approved by the Animal Experimentation Committee of the Universidad de la República.

### Animals and experimental design

Pluriparous dairy cows (n = 27, third-lactation cows, body weight (BW) = 709 ± 52.5 kg, BCS = 3.25 ± 0.25) were used in a complete randomized block design. Cows were grouped according with their expected calving date (3/22/11 ± 2.3 days n = 15), and 4/11/11 4.8 days, n = 12) blocked within groups according with BW and BCS and randomly assigned within block to one of three feeding strategies from calving (day 0) to 60 days postpartum (days):control cows fed TMR ad libitum (G0), pasture grazing with 6 h of access to paddock in one grazing session (8:00–14:00 h) and supplemented with TMR (G1), and pasture grazing with 9 h of access to paddock in two grazing sessions (8:00–14:00 and 17:00–20:00 h) and supplemented with TMR (G2). Two cows of G2 were removed from the study due to lameness thus the final treatment groups were TMR (n = 9), G1 (n = 9) and G2 (n = 7).

During the prepartum period (from −40 ± 6 days to calving) the experimental herd was managed to achieve a BCS at calving between 3.0 and 3.5 (1–5 scale; [13]). During this period, a TMR of corn silage and concentrate was fed to the cows to prevent BCS loss. During the postpartum period, all cows were assigned to experimental diets and were offered the same amount of DM (ad libitum, approximately 30.7kg DM/cow/d) only as TMR (for G0 cows) or 50 % DM as pasture and 50 % as TMR (for G1 and G2 cows). Cows (G1 and G2) grazed a second year perennial pasture of *Festuca arundinacea, Trifolium repens* and *Lotus corniculatus* (located 1.7 km from the milking parlour) in a 7-d rotational system with a mean herbage allowance of 15 kg DM/cow/d (4 cm above ground level) with 325 ± 1.8 g/kg of DM, 165 ± 1 g/kg DM of crude protein (CP), 479 ± 3.1 g/kg DM of neutral detergent fiber (NDF), 236 ± 0.5 g/kg DM of acid detergent fiber (ADF), and 1.6 Mcal/kg of net energy of lactation (NEL). The TMR, had a forage/concentrate ratio of 45/55 (DM basis) and was composed by corn or sorghum silage and a concentrate that included dry ground corn (19 %), wheat grain (12 %), soybean expeller (9 %), sunflower expeller (11 %), urea (3 %), minerals and vitamins (9 %)with a chemical composition of 544 ± 36 g/kg of DM, 158 ± 20 g/kg DM of CP, 309 ± 31 g/kg DM of NDF, 165 ± 16 g/kg DM of ADF, and 1.6 Mcal/kg of NEL. This TMR was formulated according to NRC [14] for a milk production target of 40 kg/d and a 15 % feed refusal. The TMR was offered once a day in the afternoon to G1 and G2 cows and twice a day (40 % in the morning and 60 % in the afternoon) to the G0 cows.

The proportion of TMR and pasture in the diet (DM basis) calculated for each treatment after the DM intake of TMR (based on difference between feed offered and refused) and pasture (based on alkane-dosing) was determined, indicated that diet was composed of 27 % pasture and 73 % TMR for G1 cows and 34 % pasture and 66 % TMR for G2 cows [15] Nutrient composition of estimated diets are presented in Table 1.

**Table 1:** Estimated nutrient composition of diets according to feeding strategy in early lactation

Component^a^	Treatments^b^		
	G0	G1	G2
DM, g/kg	613	532	512
CP, g/kg	156	156	159
Digestible RUP, % of DM	79.5	78.0	77.7
NDF, g/kg	336	308	301
ADF, g/kg	204	216	219
Ash, g/kg	45	62	66
NEL, Mcal/d	1.55	1.58	1.60

^a^Nutrient composition calculated from TMR and pasture DMI estimated by Fajardo et al. [15] and feed sample chemical analyses

^b^Feeding strategies from calving (day 0) to 60 days postpartum: *G0* DM offered as 100 % total mixed ration (TMR; n = 9), *G1* DM offered as 50 % pasture in one (am) grazing session (6 h) plus 50 % TMR (n = 9), *G2* DM offered as 50 % pasture in two (am/pm) grazing sessions (9 h) + 50 % TMR (n = 7)

Cows were milked twice a day (5:00 and 15:00 h), milk production was determined daily and milk samples were obtained weekly to determine fat, protein and lactose composition. Cow BCS and BW were measured weekly and blood samples were obtained weekly from −40 to +60 days immediately after the morning milking by venipuncture of the coccygeal vein in heparinized tubes. Samples were centrifuged (2000×*g* for 15 min at 4 °C) within 2 h after collection and plasma was stored at −20 °C until assayed. Liver biopsies were obtained from cows at −40 ± 6, −20 ± 3, +10 ± 4 and +55 ± 4 days using a 14-gauge biopsy needle (Tru-Core^®^-II Automatic Biopsy Instrument; Angiotech, Lausanne, Switzerland) as described by Carriquiry et al. [16]. Liver samples were immediately frozen in liquid nitrogen and stored at −80 °C until total RNA was isolated.

### Metabolite and hormone analyses

The metabolic profiles (non-esterified fatty acids(NEFA), β-hydroxybutyrate (BHB), glucose and urea) were determined by colorimetric assays on Vitalab Selectra II autoanalyzer (Vital Scientific, Dieren, The Netherlands) using commercial kits (Wako NEFA-HR(2), Wako Pure Chemical Industries Ltd., Osaka, Japan for NEFA; Randox Laboratories Limited, 55 Diamond Road, Crumlin, Country Antrim, BT29 4QY, United Kingdom for BHB; Wiener Laboratories S.A.I.C. Riobamba, Rosario, Argentina for glucose and urea). All samples were determined in the same assay for each metabolite, the intra-assay CV for all determinations was less or equal than 10 %.

Concentrations of insulin and IGF-I were measured using immunoradiometric assays (IRMA) with commercial kits (INS-IRMA; DIA Source Immune Assays S.A., Belgium and IGF-I-RIACT Cis Bio International, GIF-SUR-YVETTE CEDEX, France, respectively) previously used in bovine [17]. All samples were determined in a single assay for each hormone. For insulin, the assay detection limit was 0.7 µIU/ml, and intra-assay CV for control 1 (22.3 µIU/ml) and 2 (55.5 µIU/ml) were 8.2 and 8.3 %, respectively. For IGF-I, the assay detection limit was 0.3 ng/ml, and intra-assay CV for control 1 (41.1 ng/ml) and control 2 (521.5 ng/ml) were 7.8 and 7.9 %, respectively.

Leptin concentrations were determined by a liquid-phase radioimmunoassay (RIA) using a commercial Multi-Species Leptin kit (RIA kit, Millipore, USA) previously reported in bovines [17, 18]. The RIA had a sensitivity of 2.9 ng/ml. All samples were determined in the same assay and the intra-assay CV for control 1 (4.2 ng/ml) and control 2 (18.8 ng/ml) were 8.2 and 7.4 %, respectively. In the absence of purified bovine adiponectin, concentrations of adiponectin were measured with a human RIA kit (HADP-61 HK, Millipore, USA) using undiluted plasma samples [18, 19]. The sensitivity of the assay was 1.54 ng/ml. All samples were determined in the same assay and the intra-assay CV for control 1 (12.2 ng/ml) and control 2 (95.4 ng/ml) were 6 and 12 %, respectively.

Days to first ovulation were determined by progesterone milk concentrations twice a week. Milk was skimmed at 3000 rpm at 4 °C for 15 min. Progesterone concentrations in skim milk were measured by a solid-phase RIA using a commercial kit (Coat and Count; Diagnostic Products, Los Angeles, CA, USA). All samples were analyzed in a single assay; the sensitivity was 0.01 ng/ml, the intra-assay CV was not greater than 10.6 %. Days to first ovulation was defined as the day in which progesterone concentration in milk had two consecutive samples greater than 1 nmol/l.

### Isolation and purification of RNA

Isolation of total RNA from hepatic tissue and synthesis of cDNA by reverse transcription was performed according with Carriquiry et al. [16] (see Additional file 1). Primers (Additional file 1) to specifically amplify cDNA of target genes: *GHR*, *GHR1A*, *IGF1*, *IGF2*, *IGF binding proteins*-*1 to 6* (*IGFBP1, IGFBP2, IGFBP3, IGFBP4, IGFBP5, IGFBP6*), insulin receptor (*INSR*), long form of the leptin receptor(*LEPRb*), adiponectin receptor 1 and 2 (*ADIPOR1, ADIPOR2*), and from endogenous controls: *β*-*actin* (*ACTB*), *hypoxanthine phosphoribosyltransferase* (*HPRT*), and *ribosomal protein S9* (*RPS9*), were obtained from literature or specifically designed using the Primer3 website (http://frodo.wi.mit.edu/primer3/) based on bovine nucleotide sequences available from NCBI (http://www.ncbi.nlm.nih.gov/). Before use, primer product sizes (1 % agarose gel separation) and sequences (Macrogen Inc., Seoul, Korea) were determined to ensure that primers produced the desired amplicons.

Real time PCR reactions were performed in a total volume of 15 µl using KAPA SYBR^®^ FAST Universal 2X qPCR Master Mix (Kapa Biosystems, inc. Woburn, MA, USA) according with Astessiano et al. [20]. using the following standard amplification conditions: 10 min at 95 °C and 40 cycles of 15 s at 95 °C, 45 s at 60 °C, and 20 s at 72 °C. Dissociation curves were run on all samples to detect primer dimers, contamination, or presence of other amplicons. Each disk included a pool of total RNA from bovine liver samples analyzed in triplicate to be used as the basis for the comparative expression results (exogenous control) and duplicate tubes of water (non-template control). Gene expression was measured by relative quantification [21] to the exogenous control and normalized to the geometric mean expression of the endogenous control genes (*HPRT*, *ACTB* and *RPS9*). Expression stability of 3 selected housekeeping genes was evaluated using MS-Excel add-in Normfinder (MDL, Aarhus, Denmark). The stability values obtained with Normfinder they were 0.144, 0.121, and 0.178 for *HPRT*, *ACTB*, and *RPS9*, respectively. Amplification efficiencies or target and endogenous control genes were estimated by linear regression of a dilution cDNA curve (n = 5 dilutions, from 100 to 6.25 ng/tube; Additional file 1). Intra and inter-assay CV values were 1.9 and 4.2 %, respectively.

### Calculations and statistical analyses

Net energy (NE) and metabolizable protein (MP) calculations were based on NRC [14]. Maintenance NE requirements were calculated as NEM = 0.08 × BW^0.75^ + NEmact, where NEmact = ((((Distance/1000) × Trips) × (0.00045 × BW)) + (0.0012 × (BW)). Lactation NE requirements were calculated as NEL = milk yield × [(0.0929 × fat %) + (0.0563 × true protein %) + (0.0395 × lactose %)], using composition data derived from analysis of samples collected weekly. Metabolizable protein required for maintenance was calculated as MPM = 4.1 × (BW^0.50^) + 0.30 × (BW^0.60^) + ((DMI × 30) − [0.50 × (bacterial MP/0.8)] − bacterial MP) + endogenous MP/0.67. Metabolizable protein required for lactation (MPL) was calculated as MPL = (milk yield × true protein %)/0.67.

Estimation of the revised quantitative insulin sensitivity check index (RQUICKI) was done according to Perseghin et al. [22], i.e. RQUICKI = 1/[log(Glucose, mg/dl) + log(Insulin, μU/ml) + log(NEFA, mmol/l)], in which a low RQUICKI index indicates decreased insulin sensitivity.

Data were analyzed in a randomized block design using the SAS System program (SAS Institute Inc., Cary, NC, USA). Univariate analyses were performed on all variables to identify outliers and inconsistencies and to verify normality of residuals. Data of BCS, energy and MP balances and their components, plasma metabolite and hormone concentrations and hepatic mRNA expression were analyzed by repeated measures using the MIXED procedure with days postpartum as the repeated effect, and the appropriate covariance structure [first-order autoregressive (AR(1)) for evenly spaced data or spatial power (SP(POW)) for unevenly spaced data]. The Kenward-Rogers procedure was used to adjust the denominator degree of freedom. Data were analyzed with a model that included period (pre or postpartum), nutritional treatment within period (no treatment for period prepartum and G1, G2 and G0 for period post partum), days postpartum within period, and the interaction between treatment and days within period as fixed effects and replication and block within replication as random effects. The interaction between treatment and replication was included in the model as a random effect but as covariance parameter estimates were zero or close to zero it was removed from the model.

Days to first ovulation was defined as the number of days from calving to reinitiation of ovarian cyclicity. The probability for first ovulation was estimated by the proportion of cows with first ovulation confirmed every 5 days from day 15 to 60 postpartum. Days to first ovulation and probability of cows cycling during the first month were analyzed with a generalized lineal model using the GENMOD procedure with a model that included the fixed effect of nutritional treatment and with the Poisson and a log link or binomial distribution and a logit link specified, respectively. Tukey–Kramer tests were conducted to analyze differences between groups (α = 0.05). For all results, means were considered to differ when *P* ≤ 0.05, and trends were identified when 0.05 < *P* ≤ 0.10. Data are presented as least square means ± pooled standard errors.

## Results

### Cow productive and reproductive performance

**Fig. 1:**
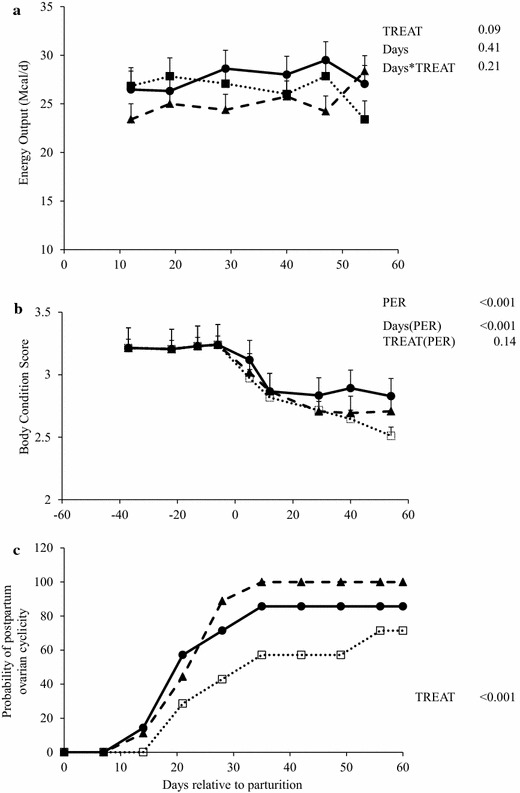
Productive responses (BCS and milk yield, (**a**) and (**b**), respectively) and probability of days to first ovulation (**c**) during peripartum and early lactation period (from −40 to 55 days relative to parturition) in pluriparous dairy cows assigned to three different feeding strategies during the postpartum. From calving (day 0) to 60 days postpartum: *G0* DM offered as 100 % total mixed ration (TMR; n = 9; *solid line*), *G1* DM offered as 50 % pasture in one (am) grazing session (6 h) plus 50 % TMR (n = 9; *dash line*), and *G2* DM offered as 50 % pasture in two (am/pm) grazing sessions (9 h) + 50 % TMR (n = 7; *dotted line*). Differences between cow groups are indicated with * when *P* ≤ 0.05. *T* treatment, *Days* day of lactation

Milk energy output tended (*P* = 0.09) to be greater for G0 than G1 cows but not for G2 cows (Table 2; Fig. 1a), whereas, estimated NEM during the first 60 days was the greatest (*P* < 0.05) for G2 and the lowest for G0 cows due to differences in NE requirement for waking and grazing activity. In contrast, milk MP requirement did not differ among treatments (Table 2). However, both estimated NEL and MP balances were greater (*P* = 0.01) for G0 than G1 and G2 cows.

**Table 2:** Effect of early feeding strategy on cow BW, BCS, and estimated net energy of lactation (NEL) and metabolizable protein (MP) balances

Variable	Treatments^1^			SE	P value
	G0	G1	G2		
Estimated NEL balance^2^					
Maintenance, Mcal of/d	10.3^c^	12.8^b^	13.9^a^	0.03	0.01
Milk, Mcal/d	27.8^x^	24.0^y^	26.5^xy^	1.25	0.09
Balance, Mcal/d	2.7^a^	−5.2^b^	−5.6^b^	1.30	0.01
Estimated MP balance^2^					
Milk, g of MP/d	1780	1620	1660	70.24	0.30
Balance, g of MP/d	0.08^a^	−0.26^b^	−0.34^b^	0.08	0.01
BW, kg	646	655	618	15	0.23
BCS, units	2.9	2.8	2.8	0.07	0.53

^1^Feeding strategies from calving (day 0) to 60 days postpartum: DM offered as 100 % total mixed ration (TMR; n = 9), *G1* DM offered as 50 % pasture in one (am) grazing session (6 h) plus 50 % TMR (n = 9), *G2* DM offered as 50 % pasture in two (am/pm) grazing sessions (9 h) + 50 % TMR (n = 7)

^2^Required NEL and MP for maintenance (including NEL for activity) and milk, values were calculated according to NRC [14]

^a,b,c^Letters denote least squares means differ (*P* ≤ 0.05)

^xy^Indicate a tendency 0.05 < *P* > 0.10, for the interaction between cow groups and treatments

Cow BCS decreased (*P* < 0.05) from −20 to +55 days and at +55 days was greater (*P* < 0.05) in G0 than G2 cows (Table 2; Fig. 1b). Days to first ovulation were greater (*P* = 0.02) for G2 than G1 cows and intermediate for G0 (21, 40 and 28 ± 5 days for G1, G2 and G0, respectively) while probability of cows cycling during the first month of lactation was greater (*P* < 0.01) for G0 and G1 than G2 cows (89, 43 and 71 % for G1, G2 and G0, respectively) (Fig. 1c).

### Plasma metabolites and hormones

**Fig. 2:**
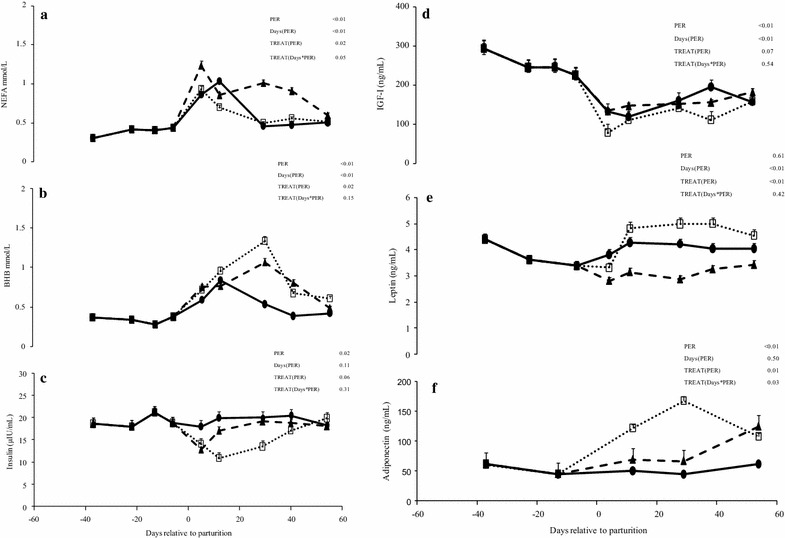
Concentrations of NEFA (**a**), BHB (**b**), insulin (**c**), IGF-I (**d**), leptin (**e**), and adiponectin (**f**) during peripartum and early lactation period (from −40 to 55 days relative to parturition) in pluriparous dairy cows assigned to three different feeding strategies during the postpartum. From calving (day 0) to 60 days postpartum: *G0* DM offered as 100 % total mixed ration (TMR; n = 9; *solid line*), *G1* DM offered as 50 % pasture in one (am) grazing session (6 h) plus 50 % TMR (n = 9; *dash line*), and *G2* DM offered as 50 % pasture in two (am/pm) grazing sessions (9 h) + 50 % TMR (n = 7; *dotted line*). Differences between cow groups are indicated with * when *P* ≤ 0.05. *T* treatment, *Days* day of lactation

Concentrations of NEFA peaked between +5 and +12 days and during the postpartum were greater (*P* = 0.02) in G1 than G2 and G0 cows, as NEFA concentrations remained elevated at +30 and +40 days only in former ones (Fig. 2a). Concentrations of BHB peaked between +15 and +30 days and during the postpartum were greater (*P* < 0.05) in G1 and G2 than G0 cows (Fig. 2b). Plasma glucose and urea were not affected by treatment, but varied during the transition and early lactation periods. Plasma glucose concentrations increased (*P* < 0.01) from calving to +55 days, while plasma urea concentrations decreased (*P* < 0.01) from −40 to calving and then increased (*P* < 0.01) until +55 days (data not shown).

Plasma insulin concentrations decreased (*P* = 0.02) around calving and during the postpartum period were greater (*P* = 0.02) in G0 than G2 cows (Fig. 2c). Plasma IGF-I concentrations decreased (*P* < 0.01) from −40 to +5 days and increased thereafter until +55 days, but prepartum concentrations were not recovered at the end of the experimental period, and during the postpartum period tended to be greater (*P* = 0.06) in G0 and G1 than G2 cows (Fig. 2d). Leptin concentrations decreased (*P* < 0.01) from −40 days to calving, increasing thereafter and during the postpartum were greater (*P* < 0.01) in G2 and G0 than G1 cows (Fig. 2e). Adiponectin concentrations increased (*P* < 0.01) from pre to postpartum and during the postpartum were greater (*P* = 0.01) in G2 than G1 and G0 cows as plasma adiponectin were elevated at +15 and +30 days only in the former ones (Fig. 2f). However, the adiponectin to leptin ratio was not affected by treatment or days of lactation (averaged 95.6, 83.3, 70.0 ± 24.4 for G1, G2 and G0 cows, respectively). The RQUICKI index decreased (*P* < 0.01) from −40 days to calving, and during the postpartum was greater (*P* = 0.02) in G2 than G1 and intermediate in G0 cows.

### Hepatic gene expression

Hepatic *GHR* mRNA was not affected by period, but tended to be affected by treatment (*P* = 0.09) being 1.8-fold greater in G1 than G2 cows during the postpartum (Table 3). However, expression of *GHR1A* decreased (*P* < 0.01) and *IGF1* mRNA tended to decrease (*P* = 0.08) from pre to postpartum while *IGF2* mRNA increased (*P* = 0.002) at +55 days for all cows (Table 3). Hepatic *IGFBP1, IGFBP4* and *IGFBP5* mRNA increased (*P* ≤ 0.04) from pre to postpartum while *IGFBP2* mRNA increased (*P* < 0.01) from −20 vs. +10 days and tended to decrease again (*P* = 0.06) at +55 days (Table 3). In contrast, *IGFBP3* mRNA tended (*P* = 0.08) to increase from −40 to −20 days and decreased (*P* < 0.01) from −20 to +10 days (Table 3). Expression of *IGFBP6* mRNA tended (*P* = 0.07) to increase from pre to postpartum and to be affected by treatment, being greater in G2 than G0 and intermediate in G1 cows during the postpartum (Table 3).

**Table 3:** Hepatic expression of genes related to the GH-IGF axis during the peripartum and early lactation periods (from −40 to 55 days relative to parturition) of dairy cows assigned to three different feeding strategies during the first 60 days of lactation

Gene^3^	Days	All^4^	Treatments^1^			SE	P value^2^		
			G0	G1	G2		PER	Days (PER)	TREAT (PER)
*GHR*							0.39	0.27	0.09
	−40	1.00	–	–	–	–			
	−20	0.88	–	–	–	–			
	10	0.75	0.78^ab^	0.91^ab^	0.56^b^	0.57			
	55	1.01	0.88^ab^	1.47^a^	0.69^b^	0.56			
*GHR1A*							<0.01	0.10	0.97
	−40	1.00^a^	–	–	–	–			
	−20	0.91^a^	–	–	–	–			
	10	0.56^b^	0.58	0.58	0.51	0.19			
	55	0.41^c^	0.42	0.40	0.41	0.18			
*IGF1*							0.08	0.70	0.20
	−40	1.02^xy^	–	–	–	–			
	−20	1.12^x^	–	–	–	–			
	10	0.84^y^	0.58	0.79	1.17	0.32			
	55	0.92^xy^	0.74	1.11	0.91	0.31			
*IGF2*							<0.01	0.05	0.91
	−40	1.00^b^	–	–	–	–			
	−20	1.20^b^	–	–	–	–			
	10	1.29^b^	1.20	1.27	1.39	0.43			
	55	1.76^a^	1.80	1.98	1.51	0.48			
*IGFBP1*							<0.01	0.42	0.25
	−40	1.00^b^	–	–	–	–			
	−20	1.15^b^	–	–	–	–			
	10	1.96^a^	2.09	1.56	2.25	0.93			
	55	2.44^a^	3.25	1.85	2.20	0.97			
*IGFBP2*							<0.01	0.10	0.94
	−40	1.00^b^	–	–	–	–			
	−20	1.24^b^	–	–	–	–			
	10	3.35^a^	3.46	3.20	3.38	0.79			
	55	2.43^a^	2.25	2.78	2.26	0.81			
*IGFBP3*							0.09	0.10	0.54
	−40	1.00^ab^	–	–	–	–			
	−20	1.25^a^	–	–	–	–			
	10	0.91^b^	1.10	0.74	0.90	0.24			
	55	1.05^ab^	1.00	1.04	1.10	0.24			
*IGFBP4*							0.04	0.54	0.73
	−40	1.00^ab^	–	–	–	–			
	−20	0.97^b^	–	–	–	–			
	10	1.14^ab^	1.05	1.04	1.32	0.41			
	55	1.35^a^	1.25	1.34	1.45	0.40			
*IGFBP5*							<0.01	0.62	0.74
	−40	1.00^b^	–	–	–	–			
	−20	1.10^b^	–	–	–	–			
	10	2.03^a^	2.09	1.60	2.41	0.84			
	55	1.75^a^	1.36	2.03	1.85	0.94			
*IGFBP6*							0.08	0.71	0.06
	−40	1.00^y^	–	–	–	–			
	−20	0.97^y^	–	–	–	–			
	10	1.13^xy^	0.93^b^	1.01^ab^	1.46^ab^	0.34			
	55	1.29^x^	0.86^b^	1.33^ab^	1.68^a^	0.34			

^1^Feeding strategies from calving (day 0) to 60 days postpartum: *G0* DM offered as 100 % total mixed ration (TMR; n = 9), *G1* DM offered as 50 % pasture in one (am) grazing session (6 h) plus 50 % TMR (n = 9), *G2* DM offered as 50 % pasture in two (am/pm) grazing sessions (9 h) + 50 % TMR (n = 7)

^2^ *PER* period (pre vs. postpartum), *TREAT* treatment. The interaction TREAT (PER) × Days was not significant (P > 0.25) for any of the genes

^3^ *GHR* = growth hormone receptor, *GHR1A* = growth hormone receptor 1A, *IGF1* = insulin-like growth factor-I, *IGF2* = insulin-like growth factor-2, *IGFBP* = IGF-binding protein 1 to 6. Relative units, data are expressed as fold difference relative to first sample (−40 days)

^4^Lsmeans for Days(PER) effect, including all treatments

^a,b,c^Letters denote least squares means differ (*P* ≤ 0.05)

^xy^Indicate a tendency 0.05 < *P* > 0.10, according to Tukey–Kramer test for the interaction between PER, Days and TREAT

Expression of *INSR* mRNA in the liver increased (*P* < 0.03) at + 55 days for all cows (Table 4). Hepatic *LEPRb* mRNA increased (*P* < 0.01) from pre to postpartum and was greater (*P* < 0.05) in G2 than G1 cows during early lactation (Table 4). Although *ADIPOR1* mRNA did not vary due to feeding strategies or days of lactation, *ADIPOR2* mRNA increased (*P* < 0.01) from pre to postpartum in all cows (Table 4).

**Table 4:** Hepatic expression of genes related to the energy metabolism during the peripartum and early lactation periods (from −40 to 55 days relative to parturition) in multiparous dairy cows assigned to three different feeding strategies during the first 60 days of lactation

Gene^3^	Days	All^4^	Treatments^1^			SE	P value^2^		
			G0	G1	G2		PER	Days (PER)	TREAT (PER)
*INSR*							<0.01	0.04	0.92
	−40	1.00^b^	–	–	–	–			
	−20	0.91^b^	–	–	–	–			
	10	1.12^ab^	1.21	1.00	1.14	0.25			
	55	1.55^a^	1.42	1.84	1.39	0.24			
*LEPRB*							<0.01	0.15	0.02
	−40	1.00^b^	–	–	–	–			
	−20	1.41^ab^	–	–	–	–			
	10	1.49^a^	1.61^abc^	1.05^c^	1.80^ab^	0.47			
	55	1.58^a^	1.55^abc^	1.17^bc^	2.03^a^	0.48			
*ADIPOR1*							0.33	0.40	0.99
	−40	1.00	–	–	–	–			
	−20	0.95	–	–	–	–			
	10	0.98	0.98	0.96	1.00	0.67			
	55	1.16	1.15	1.18	1.14	0.72			
*ADIPOR2*							<0.01	0.59	0.91
	−40	1.02^b^	–	–	–	–			
	−20	0.92^b^	–	–	–	–			
	10	2.27^a^	2.38	2.31	2.10	0.30			
	55	2.06^a^	1.83	2.26	2.09	0.30			

^1^Feeding strategies from calving (day 0) to 60 days postpartum: *G0* DM offered as 100 % total mixed ration (TMR; n = 9), *G1* DM offered as 50 % pasture in one (am) grazing session (6 h) plus 50 % TMR (n = 9), *G2* DM offered as 50 % pasture in two (am/pm) grazing sessions (9 h) + 50 % TMR (n = 7)

^2^ *PER* period (pre vs. postpartum), *TREAT* treatment. The interaction TREAT(PER) × Days was not significant (P > 0.25) for any of the genes

^3^ *INSR* = insulin receptor, *LEPRB* = full-length leptin receptor, *ADIPOR1* = adiponectin receptor 1, *ADIPOR2* = adiponectin receptor 2. Relative units, data are expressed as fold difference relative to first sample (−40 days)

^4^Lsmeans for Days (PER) effect, including all treatments

^a,b,c^Letters denote least squares means differ (*P* ≤ 0.05)

## Discussion

### Days of lactation on metabolic and endocrine profiles and liver gene expression

During the peripartum and early lactation, all cows entered a period of negative energy balance, as the rapid increase in milk yield was not met by DMI, determining various metabolic and endocrine adaptations to provide energy and nutrients to the early lactating mammary gland [3]. Cows mobilized body reserves, reflected by the loss of BCS and the elevated plasma concentrations of NEFA and BHB in blood that indicated increased lipolysis and ketogenesis [4, 9]. In addition, plasma urea decreased around calving and increased during early lactation probably, reflecting enhanced tissue protein breakdown to provide gluconeogenesis precursors as well as the increase feed intake [23].Insulin and IGF-I concentrations decreased around calving which was consistent with the reduction in DMI, characteristic of this period and with an uncoupled somatotropic axis, which mediates nutrient partitioning towards milk production [3, 5]. Indeed, the IGF-I decrease at calving was associated with *GHR1A* and *IGF1* mRNA decreases during the early postpartum while no changes were detected in the abundance of hepatic *GHR* mRNA as reported before [24]. Decreased insulin concentrations are hypothesized to be a key signal regulating this mechanism, as stimulates hepatic IGF-I secretion by modifying hepatic GHR and IGFBP secretion [25].

Most IGF-I is bound to IGFBP3 in a ternary complex, the negative energy balance would induce a shift of this complex to binary complexes in which IGF-I is bound to IGFBP1 and particularly to IGFBP2, which would in turn reduce half-life of IGF-I in blood [26]. The increased hepatic *IGFBP1* and *IGFBP2* mRNA expression and the simultaneous decrease of *IGFBP3* mRNA in all cows have been shown before [20, 27] and support this hypothesis. Indeed, Kessler et al. [28], demonstrated that as plasma IGF-I concentrations dropped during the lactation negative energy balance or during feed restriction, binding affinity of IGF-I to IGFBP1 and IGFBP2 in plasma increased while its binding affinity to IGFBP3 decreased, Moreover, *IGFBP4* and *IGFBP5* mRNA also increased during the postpartum (at +55 and +10 DPP, respectively) and it has been reported that these proteins may have also inhibitory effects on IGF-I activity [29, 30]. On the other hand, in the present study, IGF-I concentrations started to increase after the first month postpartum while *GHR1A* mRNA expression remained low and hepatic mRNA of low molecular weight *IGFBP* were still elevated, thus, other factors would be responsible for the increasing IGF-I concentrations during the second month after calving.

Interestingly, hepatic *IGF2* and *IGFBP6* mRNAs, a binding protein with high affinity to IGF-II, were low during peripartum and increased at +55 days for all cows. Relatively few studies have examined IGF-II during the transition period of the dairy cow, although its transcript is expressed at comparatively high levels in the liver [31]. The greater expression of *IGF2, IGFBP6* and also *IGFBP4* mRNA together with the greater probability of cycling after the first month postpartum is consistent a role of these genes in reproduction [32–34].

The reduced insulin and elevated NEFA concentrations during the early postpartum, determined that the in the present study RQUICKI decreased during this period, which is indicative of increased reduced insulin resistance [35]. Indeed, the adipokines adiponectin and leptin, are considered positive regulators of insulin sensitivity in various species [6, 7]. Reduced concentrations of these hormones around calving, as reported here, may increased insulin resistance of peripheral tissues, which would decrease glucose uptake by skeletal muscles and adipose tissues and increase hepatic gluconeogenesis [7], improving glucose supply to the mammary gland for enhanced milk production. Although adiponectin and leptin profiles during early postpartum period depended of the treatment (see “Discussion” below), the concentrations increased rapidly during the early postpartum. Similar findings were reported for adiponectin [36], but not for leptin [6]. Differences in leptin profiles during the postpartum between this and other reports could be the result of the restricted feeding dry period nutrition (vs. ad libitum*)* in the present study. In addition, Weber et al. [37] reported elevated leptin concentrations in dairy cows with moderate hepatic lipid fat associated with reduced abdominal fat mobilization, and in agreement with our results, with reduced insulin and NEFA concentrations and greater insulin sensitivity based on RQUICKY.

Insulin and adipokines allow for the communication of pancreas and adipose tissue with other body organs such as liver, to regulate, among others, energy metabolism and immune functions through not only alteration in blood concentrations, but also differential tissue regulation of receptor expression during the lactation cycle. Effects of leptin are mainly mediated by *LEPRb* [8] and of adiponectin via *ADIPOR1* and *ADIPOR2*, with *ADIPOR1* predominantly expressed in skeletal muscle and *ADIPOR2* in liver [7]. Consistent with the literature [8, 38], in the present study, hepatic *ADIPOR1* mRNA abundance was stable whereas *ADIPOR2* and *LEPRb* mRNA increased in early lactation, counteracting reduced adiponectin and leptin concentrations around calving. This increase in *ADIPOR2* and *LEPRb* mRNA would favor a role of these hormones on insulin sensitivity in the liver, inducing fatty acid oxidation and preventing lipid accumulation [7, 38]. Hepatic *INSR* mRNA increased during pospartum and was greater at +55 days in all cows. Our data are consistent with reports that found greater liver *INSR* mRNA in control cows than cows with fatty liver disease or ketotic dairy cows [39],which suggested that reduced expression of *INSR* mRNA in the liver during early postpartum indicates that responses to insulin are markedly decreased, which might be due to insulin resistance.

### Effect of treatment on metabolic and endocrine profile and hepatic gene expression

In the present study, although there were differences in milk NE and MP output as well as NE maintenance requirements between cow groups, the greater estimated DMI [14], thus, the greater estimated NEL and MP intakes, would explain differences in NE and MP balances between G0 and grazing cows (G1 and G2). The NE and MP balances indicated that grazing cows (G1 and G2 cows) mobilized greater amounts of tissue which was partially reflected in different BCS at +55 days between G2 and G0 cows.

Nevertheless, plasma NEFA concentrations in early lactation did not differ between G0 and G2 cows and were less than in G1 cows. The reduced NEFA and BHB concentrations in G0 cows would indicate a low rate of lipolysis and hepatic ketogenesis in agreement with their better energy balance [9]. In contrast, the reduced NEFA concentrations in G2 cows (when compared to G1 cows)that were accompanied with elevated BHB concentrations (not different than the observed in G1 cows), could probably be related to a differential tissue utilization of NEFA due to the increased walking and grazing activities in these cows. Plasma NEFA fuel, through mitochondrial fatty acid oxidation and Krebs cycle, energy production in muscle for muscle contraction or exercise, [3, 4]. Indeed, it has been suggested that increased walking activity could reduce plasma NEFA in dairy cows [40]. In addition, the greater plasma BHB in grazing cows (G1 and G2) could not only be related to an increased hepatic ketogenesis but also could reflect an increased synthesis of butyric acid in the rumen as fresh pastures were included in the diet [41]. The greater plasma insulin observed in G0 than G2 cows and IGF-I concentrations in G0 and G1 than G2 cows during the postpartum, was consistent with the greater non-fiber carbohydrate concentration, the reduced energy requirements for activity and the greater estimated energy balance in the former ones [10, 42].

In the present study, the lowest concentrations of insulin and NEFA in G2 cows determined an increased RQUICKI in this group indicative of greater insulin sensitivity [35]. Adiponectin and leptin, which have insulin sensitizing actions, were the greatest in G2 cows during early lactation. Circulating adiponectin concentrations, insulin sensitivity, and basal fat oxidation rate have been showed to increase with exercise in humans [43]. Therefore, greater walking and grazing activity because of the increased number of sessions (two grazing sessions) and access time to pasture (9 h) in G2 cows may explain these results.

We found and elevated expression of hepatic *IGFBP6* mRNA in G2 cows consistent with Fenwick et al. [27] that highlight a role for this binding protein in undernutrition or energy deficiency. Moreover, Cummins et al. [44] reported that cows with lower fertility had greater abundance of *IGFBP6* mRNA in the liver during the transition period in comparison with higher fertility cows. In agreement with the latter result, days to first ovulation were the longest and probability of cycling cows was the lowest in G2 cows. Indeed, Meikle et al. [9] reported that although there were no differences in milk production, dairy cows grazing medium sward herbage allowance showed a delayed reinitiation of ovarian cyclicity and lower BCS postpartum when compared to TMR and high herbage allowance grazing cows.

## Conclusion

In this work, NE, but not MP, demands for maintenance and/or milk increased in G2 cows when compared with G1 and G0 cows, respectively. However, both NE and MP balances were less in grazing cows (G1 and G2) than in G0 cows. Metabolic-endocrine profiles of cows with different feeding strategies in early lactation reflected not only changes in milk energy output and energy balance but also in walking and grazing activity. Concentrations of insulin and IGF-I were increased in G0 cows whereas plasma adiponectin and both, insulin and leptin sensitivity were improved G2 cows. The greater energy demands and/or reduced energy balance in G2 cows when compared to both G1 and G0 cows implied a metabolic stress that impacted negatively on reproductive function (delayed reinitiation of ovarian cyclicity and reduced probability of cycling cows).

## Additional file

10.1186/s13028-015-0163-6 Primers used for the quantification of target and endogenous control gene cDNA.
