# Supplementary material for: Metabolic and endocrine profiles and hepatic gene expression of Holstein cows fed total mixed ration or pasture with different grazing strategies during early lactation
Source: Acta Vet Scand. 2015 Oct 16;57:70. doi: 10.1186/s13028-015-0163-6 (PMC4609040; doi:10.1186/s13028-015-0163-6)
Supplement: Supplementary file 1 — 10.1186/s13028-015-0163-6 Primers used for the quantification of target and endogenous control gene cDNA. [file 13028_2015_163_MOESM1_ESM.doc]

**Additional file 1** Primers used for the quantification of target and endogenous control gene cDNA

| Gene^1^ | Accession  no.^2^ |  | Primer sequence | Length  (bp) | Efficiency |
| --- | --- | --- | --- | --- | --- |
| *GHR* | NM_176608 | Sense | TCTGGGAATCCTAAATTCACCAA | 91 | 1.09 |
|  |  | Antisense | CTGTAAACTGTGATTAGCCCCATCT |  |  |
| *GHR 1A* | AY748827 | Sense | AGCCTGGAGGAACCATACGA | 94 | 1.33 |
|  |  | Antisense | GCTGCCAGAGATCCATTCTGTA |  |  |
| *IGF1* | XM_612412 | Sense | CCAGACAGGAATCGTGGATG | 89 | 1.27 |
|  |  | Antisense | ACTTGGCGGGCTTGAGAG |  |  |
| *IGF2* | NM_174087 | Sense | TTG CAG GTA GGC TTG TCC TT | 98 | 1.09 |
|  |  | Antisense | CAG GTT TGG GTC TTT GGT GT |  |  |
| *IGFBP1* | NM_174554 | Sense | TAC AGA AGT GGA AGG AGC CCT | 127 | 1.21 |
|  |  | Antisense | AAT CCA TTC TTG TTG CAG TTT |  |  |
| *IGFBP2* | NM_174555 | Sense | ATGCGCCTTCCGGATGA | 83 | 1.15 |
|  |  | Antisense | GTTGTACAGGCCATGCTTGTCA |  |  |
| *IGFBP3* | NM_174556 | Sense | AGCACAGACACCCAGAACTTCT | 86 | 1.19 |
|  |  | Antisense | TTCAGCGTGTCTTCCATTTCC |  |  |
| *IGFBP4* | NM_174557 | Sense | ARG TGC CTG ATG GAG AAA GG | 145 | 0.98 |
|  |  | Antisense | AAG GCA GAG CCA CAG ACA GT |  |  |
| *IGFBP5* | NM_1105327 | Sense | CAA GCC AAG ATC GAA AGA GAC T | 86 | 1.01 |
|  |  | Antisense | AAG ATC TTG GGC GAG TAG GTC T |  |  |
| *IGFBP6* | NM_1040495 | Sense | GGA GAG AAT CCC AAG GAG AGT AA | 100 | 0.97 |
|  |  | Antisense | GAG TGG TAG AGG TCC CCG AGT |  |  |
| *INSR* | XM_590552.4 | Sense | CTGAAGCCAAGGCAGATGATATT | 77 | 0.90 |
|  |  | Antisense | GCCACATCAAGTGAACAACGTT |  |  |
| *LEPRb* | AB199589 | Sense | ACCACACCTTCCGTTCTCAG | 164 | 1.08 |
|  |  | Antisense | GGGACAACACTCTTGACTC |  |  |
| *ADIPOR1* | NM_1034055 | Sense | GGC TCT ACT ACT CCT TCT AC | 154 | 1.04 |
|  |  | Antisense | ACA CCC CTG CTC TTG TCT G |  |  |
| *ADIPOR2* | NM_1040499 | Sense | GGC AAC ATC TGG ACA CAT C | 203 | 0.98 |
|  |  | Antisense | CTG GAG ACC CCT TCT GAG |  |  |
| *ACTB* | BT030480 | Sense | CGTGGC TACAGCTTCA CC | 53 | 1.15 |
|  |  | Antisense | GAA ATCGTCCGTGACATCAA |  |  |
| *HPRT* | XM_580802 | Sense | TGGAGAAGGTGTTTATTCCTCATG | 105 | 1.05 |
|  |  | Antisense | CACAGAGGGCCACAATGTGA |  |  |
| *RPS9* | NM_1101152 | Sense | CCT CGA CCA AGA GCT GAA G | 63 | 1.03 |
|  |  | Antisense | CCT CCA GAC CTC ACG TTT GTT C |  |  |

^1^*GHR* = growth hormone receptor, *GHR1A* = growth hormone receptor 1A*, IGF1* = insulin-like growth factor-I, *IGF2* = insulin-like growth factor-II, ***IGF binding proteins-1 to 6 (IGFBP1, IGFBP2, IGFBP3, IGFBP4, IGFBP5, IGFBP6)***, *INSR* = insulin receptor, *LEPRb* = full-length leptin receptor, ***ADIPOR1, ADIPOR2, and*** *ACTB* = ß-actin, *HPRT* = hypoxanthine phosphoribosyltransferase **and *RPS9 =*** ***ribosomal protein S9* as an** endogenous control gene.

^2^GeneBank bovine sequences.
